# Determinants of Mortality among Cervical Cancer Patients Attending in Tikur Anbessa Specialized Hospital, Ethiopia: Institutional-Based Retrospective Study

**DOI:** 10.1155/2021/9916050

**Published:** 2021-06-17

**Authors:** Mulugeta Wassie, Beletech Fentie, Tseganesh Asefa

**Affiliations:** ^1^School of Nursing, College of Medicine and Health Sciences, University of Gondar, Gondar, Ethiopia; ^2^Department of Pediatrics and Child Health Nursing, School of Nursing, College of Medicine and Health Sciences, University of Gondar, Gondar, Ethiopia

## Abstract

**Background:**

Globally, about 570,000 cases and 311,000 deaths of cervical cancer occurred in 2018. It was the leading cause of cancer-related deaths among women in Africa. The global mean age at death of cervical cancer was about 59 years. This study aimed to assess the determinants of cervical cancer mortality among cervical cancer patients attending in Tikur Anbessa Specialized Hospital (TASH).

**Methods:**

Institutional-based retrospective cohort study was conducted in the oncology center of TASH, Ethiopia, from March to April 2019. Data were extracted from patients' chart using structured checklist and analyzed using Stata 14.2. Cox regression was used to identify variables that affect the outcome variable.

**Result:**

From the total of 2045 reviewed medical records of cervical cancer patients, 1057 medical records were found to be complete and included in this study. The incidence of mortality among cervical cancer patients was 15.6/100/years. Mortality was significantly increased with advanced age (adjusted hazard ratio (AHR) = 1.02, 95% CI (1.01–1.03)), comorbidity (AHR = 1.8, 95% CI (1.39–1.89)), being anemic (AHR = 1.42, 95% CI (1.07–1.89)), advanced stage (AHR = 1.63, 95% CI (1.24–2.13)), and being substance user (AHR = 2.71, 95% CI (2.08–3.53)).

**Conclusion:**

The study revealed that the incidence of mortality within the cohort was 15.6/100/years. Mortality was significantly increased with advanced age, anemia, advanced stage, comorbidity, and using substances. It is better to give special attention to patients with anemia, advanced age, advanced stage, comorbidity, and substance usage. In addition, expanding cervical cancer early screening will decrease the mortality of patients.

## 1. Background

Cancer of uterine cervix was graded in the top three malignancies, affecting females with age less than 45 years. Worldwide, nearly 570,000 cases and 311,000 deaths of cervical cancer occurred in 2018. The estimated incidence of the disease was 13.1/100,000 and it varied broadly among countries, with rates ranging from 2 to 75/100,000 [1]. It is the main reason of cancer-related mortality among females in Africa. The mean age at diagnosis was 53 years, fluctuating from 44 to 68 years globally, and the average age at death was 59 years, ranging from 45 years to 76 years [1, 2].

The incidence and mortality rates of cervical cancer are extremely higher in regions with low and median human development index (HDI) as compared to those with very high and high HDI; indeed, death and incidence are more concentrated in low HDI countries [3, 4].

Early screening services of cervical cancer are changing from cytology-based to implementation of molecular screening in economically advanced regions of the world. In contrast, economically poor countries with greater magnitude of the disease continue to face monetary and logistic constraints to utilize early screening and human papillomavirus vaccine. Low-cost screening modalities, practical strategies, discounting cost of procurement, and delivery approaches for human papillomavirus vaccine shall be realized in low resource countries [5–7].

Regional indicators of cervical cancer are scarce in many Sub-Saharan Africa countries due to scarcities of systematic reporting and cancer registries. It may also be challenging to raise this disease as a priority problem if local actors are not aware of the condition of their home countries [6, 8–10].

Regular screening is linked with strong reduction in cervical cancer mortality and morbidity. It is expected that early screening of cervical cancer prevents 70% of cervical cancer deaths in all ages, whereas, if every women regularly attend cervical cancer screening, nearly 83% of deaths could be prevented in the females of age 35–64 years [11]. Vaccination against human papillomavirus and regular screening should be introduced and expanded to decrease morbidity and subsequent costs in both human lives and other resources [12]. In addition, early diagnosis of cervical cancer and prevention of anemia in cervical cancer patients can reduce the mortality rate of the disease [13, 14]. Since there is limitation of data about determinants of mortality among cervical cancer patients in Ethiopia, this research will fill the gaps and be used as the base line information by health policy makers and administrators in the country.

## 2. Methods and Materials

### 2.1. Study Design, Period, and Area

Institutional-based retrospective cohort study was conducted in the oncology center of Tikur Anbessa Specialized Hospital (TASH), Ethiopia, from March to April 2019. TASH is the biggest referral public hospital in Ethiopia which was established in 1972. It is the training center of health professionals with undergraduate and postgraduate programs. It has over 800 beds and the beds reserved for cancer care are 20. The hospital is the only oncology center in the country and used as the only cancer registry center in Addis Ababa city.

### 2.2. Populations of the Study

Medical records of women diagnosed with cervical cancer in TASH oncology center were source populations and all medical records of cervical cancer patients from January 1, 2014, to December 31, 2018, were study populations. Medical records with incomplete information in addition to those not found during data collection period were excluded.

### 2.3. Sample Size and Sampling Procedures

The sample size of this study was all cervical cancer patients' medical records recorded from January 2014 to December 2018. Profiles of patients diagnosed between January 2014 and December 2018 in the TASH were assessed and 1057 charts that fulfilled the inclusion criteria were identified and data were collected from them. Death of cervical cancer patients was the dependent variable and sociodemographic characteristics (marital status, residential address, age at diagnosis, substance use, number of children, region, and occupation) and pathological and clinical factors (baseline anemia, histology type, stage, comorbidity, and types of comorbidities) were independent variables.

### 2.4. Operational Definitions

Stage at diagnosis: the revised FIGO staging for carcinoma of the vulva, cervix, and endometrium was used in this study [15].

  Anemia: patients' hemoglobin level below 12.0 g/dl was classified as anemic [16].
  Comorbidity: It is the presence of any conditions (mentioned in the Carlson Comorbidity Index [17]) other than cervical cancer at diagnosis.
  Substance use: patients who used one, two, or all of the three substances (cigarate, chat, and alcohol) were considered [18].

### 2.5. Data Collection Tools and Quality Assurance

Data were collected from patients' charts using pretested and structured checklist prepared. The checklist consisted three parts: sociodemographic factors, pathological factors, and clinical factors. Two supervisors with second degree in oncology nursing and three data collectors with first degree in nursing were involved in the data extraction process. To maintain the quality of the data, one day intensive training was given to supervisors and data collectors. In addition, pretest of the checklists was conducted by considering 5% of the total sample size to test its consistency with actual data collection and necessary corrections were made accordingly.

### 2.6. Data Processing and Analysis

Data were coded and then entered, edited, and cleaned using EpiData 3.1 and exported to Stata 14.2 statistical software for analysis. Frequencies, proportions, and descriptive statistics were used to describe the study population in relation to relevant variables.

Incidence of mortality was calculated for the entire study period. Cox proportional hazards regression was used to analyze independent variables affecting the outcome variable. Variables with a significance level below 0.2 in the bivariable regression were included in a multivariable regression analysis. Variables in multivariable regression with *p* value <0.05 were considered to have significant interference with the outcome variable (mortality) with 95% confidence level.

### 2.7. Ethical Clearance

Ethical approval for this study was obtained from the Institutional Review Board of School of Nursing and Midwifery, Addis Ababa University. The letter of permission was written from the School of Nursing and Midwifery to the oncology center of TASH. Then, the cancer center chief administrator allowed us to collect the data from the cervical cancer patents medical records. The study was conducted without individual informed consent since data collection process was fully relied on chart review.

## 3. Results

### 3.1. Sociodemographic Characteristics of Cervical Cancer Patients

Initially, 2045 medical records of cervical cancer patients were reviewed and 1057 medical records were found to be complete and included in this study. The mean age of the patients was 49.5 years with SD of ±0 11.8. About 58% patients came from urban area. Nearly one-third of the patients come from Oromia reginal state and about 85.6% were unemployed. Nearly 63% of the patients were married; 17% were not substance users. Around 42% of the patients had more than three children and 3.78% had no children (Table 1).

### 3.2. Clinical and Treatment-Related Characteristics of Cervical Cancer Patients

About 57% of the patients were diagnosed at late stage (stages III and IV). Nearly one-third of the patients had comorbidity and HIV/AIDS was the most (55.9%) prevalent comorbidity type. Nearly half (51.18%) of the patients were anemic and approximately 91% of the patients had squamous cell carcinoma. About half (49.2%) of the patients received radiation therapy, and from these, about 59% patients received palliative radiation therapy (Table 2).

### 3.3. Incidence of Mortality among Cervical Cancer Patients

The incidence of mortality among cervical cancer patients was 15.60 (95% (13.96–17.45)) per 100 person-years with 3.7 years (44.4 months) of median survival time. The overall survival was 54.8% in the five years of follow-up period. The five-year survival of patients with late and early stages was 44.93% and 69.89%, respectively, whereas the five-year survival of patients with advanced and early age was 38.95% and 85.86%. About 30.47% and 61% of substance users and nonusers, 40.34% and 73.45% of anemic and nonanemic patients, 36.24% and 65.62% comorbid and noncomorbid patients, respectively, survived at least for five years.

About 43.9% of patients within age group of ≥60, but only 7.61% of patients with the age group ≤30, had died. Nearly 30% of rural dwellers, 30% of unemployed, 53% substance users, and 85% of patients without children died in the current study (Table 1). Nearly 38% of the patients with late stage (stages III and IV) and about half (46.8%) of the patients with comorbidity, 42.5% with anemia, 33% treated with chemo and radiation therapy, and 37% patients who got palliative radiation therapy had died in this cohort (Table 2).

### 3.4. Determinants of Mortality among Cervical Cancer Patients

Independent variables were individually regressed against the outcome variable and the variables with the *p* value <0.2 were included in the multivariable Cox regression. In bivariable analysis, age, comorbidity, anemia, stage, substance, and aim of radiation therapy were associated with the outcome variable at *p* value <0.2. But in multivariable analysis, age, substance, anemia, stage of cancer, and comorbidity were significantly associated with the outcome variable at *p* value <0.05 with 95% CI.

Aged patients had died about 1.02 (95% CI (1.01–1.03)) times more than their counterparts. Patients with comorbidity died 1.80 (95% CI (1.39–1.89)) times more than those without comorbidity and anemic patients died 1.42 (95% CI (1.07–1.89)) times more than nonanemic patients.

Study patients with advanced stage died 1.63 (95% CI (1.24–2.13)) times more than with early stages, whereas substance users died 2.71 (95% CI (2.08–3.53)) times more than nonusers (Table 3).

## 4. Discussion

The current study aimed to identify determinants of mortality among cervical cancer patients attending in Tikur Anbessa Specialized Hospital oncology center. The incidence rate of mortality among cervical cancer patients was 15.6 per 100 person-years in the current study. This finding is lower than the study conducted in Brazil with mortality rate of 18.9% [19], but in line with Nigerian finding with 14.8% incidence rates of mortality [20]. The disagreement with Brazilian finding might be due to socioeconomic differences of the study participants, study period, and sample size differences.

The current study revealed that aged cervical cancer patients' mortality was higher than their counterparts. This is consistent with the study conducted in China [21]. This similarity could be due to the fact that as age increases, cervical cancer survival decreases [22, 23]. But the finding is inconsistent with the study conducted in Colombia, which revealed that more cervical cancer patients died in the age groups of 20 and 49 due to young families' economic status [24]. This difference might be due to study setting, study time, and socioeconomic differences of the study participants.

Cervical cancer patients with comorbidity had died about two times more than those without comorbidity. This finding is supported by the studies conducted in Australia [25], New Zealand [26], and Italy [27]. This consistency could be as comorbidities can limit the opportunities of the treatment options, increase the risk of treatment complications, and related with discontinuation or nonadherence of treatment [28]. Furthermore, comorbidities decrease the cancer treatment effectiveness and increase comorbidity-associated death [17].

Patients who were anemic died more than nonanemic patients. This finding is supported by studies done in Nigeria [29] and Ethiopia [30]. This could be as anemia is more associated with hypoxia, which reduces tumor sensitivity to antiblastic treatments and increase mortality [31, 32].

Patients who presented with advanced stage died more than those with early stage. This result is in line with the studies conducted in New Zealand [33, 34]. The increment of mortality could be as the cancer stage getting advanced, its metastasis rate could be increased and these factors might decrease the probability to respond a treatment and lead to death [35].

Substance users in the current study died about three times more than nonusers. The possible justification of this finding could be as substances residue in cervical mucosa lead to damage in the DNA of cervical cells and these make the immune system less effective to fight against cervical cancer [36–38].

## 5. Limitations

Even though this study involved relatively large sample size, cause-specific mortality incidence was not determined due to lack of data on specific cause of death. This may overestimate the incidence of mortality rate. Information such as educational level, history of abortion, and age at first marriage were not recorded and not included in the study. Finally, the information was not recorded for the purpose of research that caused many chars with incomplete information.

## 6. Conclusion

The study revealed that the incidence of mortality among cervical cancer patients was 15.6 per 100 person-years. Mortality of cervical cancer patients was significantly increased with being advanced age, anemic, advanced stage, and comorbid and using substances. It is better to give special attention to patients with anemia, advanced age, advanced stage, comorbidity, and substance usage. Cervical cancer early screening and educating patients about bad effects of using substances can decrease mortality.

## Figures and Tables

**Table 1:** Sociodemographic characteristics of cervical cancer patients and mortality related to these characteristics in TASH.

Variables	Category	Response	Mortality	
			Yes	No
		*N* (%)	*N* (%)	*N* (%)
*Age*	<30	92 (8.70)	7 (7.61)	85 (92.39)
	30–49	219 (20.72)	36 (16.44)	183 (83.56)
	41–49	262 (24.79)	70 (26.72)	192 (73.28)
	50–59	245 (23.18)	91 (37.14)	154 (62.86)
	≥60	239 (22.61)	105 (43.93)	134 (56.07)

*Residence*	Urban	614 (58.09)	176 (28.66)	438 (71.34)
	Rural	443 (41.91)	133 (30.02)	310 (69.98)

*Region*	Amhara	281 (26.58)	94 (33.45)	187 (66.55)
	Oromia	341 (32.26)	97 (28.45)	244 (71.55)
	Tigray	30 (2.84)	10 (33.33)	20 (66.67)
	SNNP	95 (8.99)	33 (34.74)	62 (65.26)
	Addis Ababa	280 (26.49)	68 (24.29)	212 (75.71)
	Others	30 (2.84)	7 (23.33)	23 (76.67)

*Occupation*	Unemployed	905 (85.62)	275 (30.39)	630 (69.61)
	Employed	152 (14.38)	34 (22.37)	118 (77.63)

*Marital status*	Single	392 (37.09)	111 (28.32)	281 (71.68)
	Married	665 (62.91)	198 (29.77)	467 (70.23)

*Substance*	User	182 (17.22)	97 (53.30)	85 (46.70)
	Nonuser	875 (82.78)	212 (24.23)	663 (75.77)

*Children*	None	40 (3.78)	34 (85.00)	16 (15.00)
	One	69 (6.53)	24 (34.78)	45 (65.22)
	Two	147 (13.91)	49 (33.33)	98 (66.67)
	Three	361 (34.1)	122 (33.80)	239 (66.20)
	More than 3	440 (41.63)	108 (24.55)	332 (75.45)

**Table 2:** Clinical and treatment-related characteristics and mortality related to these characteristics among cervical cancer patients in TASH.

Variables	Category	Response	Mortality	
			Yes	No
		*N* (%)	*N* (%)	*N* (%)
*Stage*	Early	457 (43.24)	81 (17.72)	376 (82.28)
	Late	600 (56.76)	228 (38.00)	372 (62.00)

*Comorbidity*	Yes	344 (32.54)	161 (46.80)	183 (53.20)
	No	713 (67.46)	148 (20.76)	565 (79.24)

*Types of comorbidities*	HIV	194 (55.91)	99 (51.03)	95 (48.97)
	Hypertension	79 (22.77)	32 (40.51)	47 (59.49)
	Others	74 (21.33)	31 (41.89)	43 (58.11)

*Anemia*	Yes	541 (51.18)	230 (42.51)	311 (57.49)
	No	516 (48.82)	79 (15.31)	437 (84.69)

*Histopathology*	Squamous cell	959 (90.73)	279 (41.89)	680 (70.91)
	Adenocarcinoma	98 (9.27)	30 (30.61)	68 (69.39)

*Treatment initiated*	Sur and chemo	17 (1.60)	4 (23.53)	13 (76.47)
	Chemo	19 (1.80)	3 (15.79)	16 (84.21)
	Radiotherapy	520 (49.2)	147 (28.27)	373 (71.73)
	Chemo and RT	375 (35.4)	125 (33.33)	250 (66.67)
	Sur and RT	111 (10.5)	23 (20.72)	88 (79.28)
	Chemo + Sur + RT	15 (1.4)	7 (46.67)	8 (53.33)

*Aim of RT*	Radical	388 (36.71)	75 (19.33)	313 (80.67)
	NO RT	46 (4.35)	5 (10.87)	41 (89.13)
	Palliative	623 (58.94)	229 (36.76)	394 (63.24)

Note: Sur = surgical treatment; chemo = chemotherapy; RT = radiation therapy.

**Table 3:** Results of bivariable and multivariable Cox regression analysis of cervical cancer patients at TASH, Ethiopia (*n* = 1057).

Variables	Category	CHR (95% CI)	*p* value	AHR (95% CI)	*p* value
*Age*		1.01 (1.00–1.02)	0.007^*∗*^	1.02 (1.01–1.03)	0.001^*∗∗*^

*Comorbidity*	Yes	2.51 (2.01–3.14)	<0.001^*∗*^	1.80 (1.39–1.89)	<0.001^*∗∗*^
	No	1.0		1.0	

*Anemia*	Yes	2.05 (1.59–2.66)	<0.001^*∗*^	1.42 (1.07–1.89)	0.015^*∗∗*^
	No	1.0		1.0	

*Stage*	Early	1		1.0	
	Advanced	2.03 (1.57–2.61)	<0.001^*∗*^	1.63 (1.24–2.13)	<0.001^*∗∗*^

*Substance*	User	3.72 (2.92–4.75)	<0.001^*∗*^	2.71 (2.08–3.53)	<0.001^*∗∗*^
	Nonuser	1.0		1.0	

*Aim of RT*	Radical	1.0		1.0	
	No radiation	0.55 (0.23–1.39)	0.209	0.58 (0.23–1.45)	0.247
	Palliative	1.82 (1.40–2.37)	<0.001^*∗*^	1.17 (0.88–1.55)	0.279

Note: ^*∗*^Variables associated with the outcome variable in bivariable analysis; ^*∗∗*^variables significantly associated with the outcome variable in multivariable analysis at 95% level of significance (*p* < 0.05); 1 = reference category.

## Data Availability

Data will be available upon reasonable request from the corresponding author.

## Abbreviations

AHR: Adjusted hazard ratio
CHR: Crude hazard ratio
HIV: Human immunodeficiency virus
FIGO: International Federation of Gynecology and Obstetrics
TASH: Tikur Anbessa Specialized Hospital.
